# Vault changes and pupillary responses to light in myopic and toric implantable collamer lens

**DOI:** 10.1186/s12886-021-02119-7

**Published:** 2021-10-16

**Authors:** Ying Xiong, Yingyan Mao, Jing Li, Xiuhua Wan, Meng Li, Jingshang Zhang, Jinda Wang, Xiuli Sun

**Affiliations:** 1 Department of Ophthalmology, Beijing Tongren Eye Center, Beijing Tongren Hospital, Capital Medical University, No.1 Dongjiaomin Lane, Dongcheng District, BeiJing, 100,000 China; 2 Beijing Institute of Ophthalmology, Beijing Tongren Eye Center, Beijing Tongren Hospital, Capital Medical University, Beijing, China; 3 Beijing Advanced Innovation Center for Big Data-Based Precision Medicine, Beihang University & Capital Medical University, Beijing Tongren Hospital, Beijing, China

**Keywords:** Implantable collamer lens, High myopia, Vault, Pupillary response

## Abstract

**Background:**

Achieving an appropriate vault is the main concern after the implantation of Implantable Collamer Lens (ICLs) for surgical correction of high myopia. The vault will vary with time and optical parameters, such as accommodation and pupil size. This research is to evaluate the vault change in Myopic and Toric ICLs under different lighting conditions; and to analyze the relationship between vault changes and pupillary responses to light.

**Methods:**

We enrolled and analyzed 68 eyes from 68 patients who were implanted with Myopic EVO ICLs; we also included 60 eyes from 60 patients who were implanted with Toric EVO ICLs. The anterior chamber depth, pupil size and the post-operative vault were evaluated, 1 week after the operation, using a Visante Optical Coherence Tomography (OCT) under different lighting conditions. For each eye that was assessed, we calculated the vault change, which is defined as the difference between vault under mesopic condition and photopic condition; and the rate of vault change, which is defined as the vault change divided by mesopic vault.

**Results:**

No significant difference was noted with the anterior chamber depth between mesopic and photopic conditions in either group. A significant decrease in vault and pupil size was detected under photopic condition in both groups. We found no difference in vault change between Myopic and Toric EVO ICLs under different lighting conditions. Moreover, the rate of vault change had a significant decrease with increased mesopic vault (baseline value).

**Conclusions:**

Too low a mesopic vault has a big rate of vault change, which may cause the contact of ICL with crystalline lens in photopic state; Too high a mesopic vault would constrict the posterior movement of pupil. The findings of the study suggest that, for patients with high or low vault, we should be more careful and must perform checks in different lighting conditions.

## Introduction

The Implantable Collamer Lens (ICLs) is often the first choice for surgical correction of high myopia [1, 2]. Achieving an appropriate vault is the main concern after the implantation of ICL [3]. At present, the traditional vault measurement methods are static and are performed in a fixed environment. However, many studies show that the degree of vault will vary with time and optical parameters, such as accommodation and pupil size [4–7].

When accommodation occurs, the pupil contracts, the suspensory ligament relaxes, the ciliary muscle contracts, and the anterior surface of the natural lens becomes convex. The factors that induce accommodation can be divided into dynamic and static types. Dynamic accommodation is induced by continuously increasing short-distance visual target stimulation. Studies show that the natural lens bulges by 400 μm and its thickness increases by 600 μm in normal adults after 10D accommodation. Every 0.2 mm forward movement of the natural lens can induce the 6D accommodation force, that is, for every 1D accommodation force, the natural lens moves forward by 0.03 mm [8]. Lee et al. [9] believes that the change of vault caused by accommodation mainly lies in the forward movement of the natural lens and the change of its movement. Static accommodation can induce maximal accommodation with the help of medicine. After Sanders et al. [10] used pilocarpine to constrict the pupil, the distance from the posterior surface of the ICL to the anterior capsule of the lens was significantly reduced compared to the non-accommodated state, while the distance from the corneal endothelium to the anterior surface of the ICL changed by a very small margin, which indicated that it was mainly the increased iris tension under the drug-induced accommodation that hindered the anterior movement of the ICL.

Besides accommodation, pupillary response to light could induce a highly adaptive ICL posterior movement which will result in decreased vault. This study intends to investigate and interpret the vault change under different lighting conditions and the relationship between vault and pupillary responses to light after myopic and toric EVO ICL (Implantable Collamer Lens with a central port, V4c) implantation. Moreover, by quantitatively analyzing the movement of EVO ICL relative to the lens due to changes in lighting conditions, the rate of vault change was introduced to analyze the relationship between vault and pupillary responses to light.

### Patients and methods

#### General data

The subjects were high myopia patients who received EVO ICL implantation in Beijing Tongren hospital from August 2019 to December 2019.

Inclusion criteria: Aged 18–45 years; No history of intraocular and corneal refractive surgery except ICL implantation; The clinical data shows a complete record, the subject was informed of the study and they signed the informed consent form.

Exclusion criteria: Complications and dysfunctional vital organs or serious systemic diseases; Pregnant or lactating women; Anterior chamber depth of<2.6 mm; Glaucoma or high intraocular pressure; History of non-myopic fundus degeneration or macular disease.

One hundred twenty-eight eyes (first operated) from 128 patients (100 women, 28 men) who were implanted with the EVO ICL for myopia correction were included in this study; 68 eyes were implanted with the myopic ICL and 60 eyes with the toric ICL. All procedures carried out in this study were in accordance with the ethical standards of the institutional and national responsible committee on human experimentation and the Helsinki Declaration of 1975 and its later amendments or equivalents.

#### ICL implantation

The implants for EVO ICL implantation in all the patients were monolithic EVO ICL made by Staar, an American company. The diameter of the central optical zone is 4.65 ~ 5.50 mm, and the overall diameter (OD) of the lens comes in 12.1 mm, 12.6 mm, 13.2 mm and 13.7 mm sizes. The range of the lens power (P) is − 3.0 ~ − 23.0D. The diopter of EVO ICL is calculated, via software that is provided by Staar, based on the corneal curvature, corneal thickness and ACD of patients. The length of the EVO ICL is slightly longer than the corneal transverse diameter to ensure that a certain vault is maintained after the EVO ICL implantation. The indications were strictly evaluated before the operation, and a preoperative ophthalmic examination was performed. The EVO ICL implantation was performed by the same doctor with the assistance of a 20G perfusion head. Compound Tropicamide Eye Drops (Santen Pharmaceutical Co., Ltd., Japan) were used, 0.5 h (30 min) before the operation, for pupil dilation. Topical anesthesia was administered to the operated eye with Proparacaine Hydrochloride Eye Drops (Alcaine, USA), and routine disinfection and draping was performed. An anterior chamber puncture was performed at 6 o’clock of corneal limbus, and a 20G perfusion head of anterior chamber maintainer was fixed in place. For the supratemporal limbus, a 3 mm clear corneal tunnel incision was made to gain access into the anterior chamber, and a V4c ICL was injected into the anterior chamber with an injector. The V4c’s four loops were adjusted to the posterior chamber, the anterior chamber maintainer was then taken out with the incision being subjected to a watertight treatment-the eye was finally bound up. Routine antibiotic and glucocorticoid eye drops were administrated after the operation.

#### Measurement parameter

**Fig. 1:**
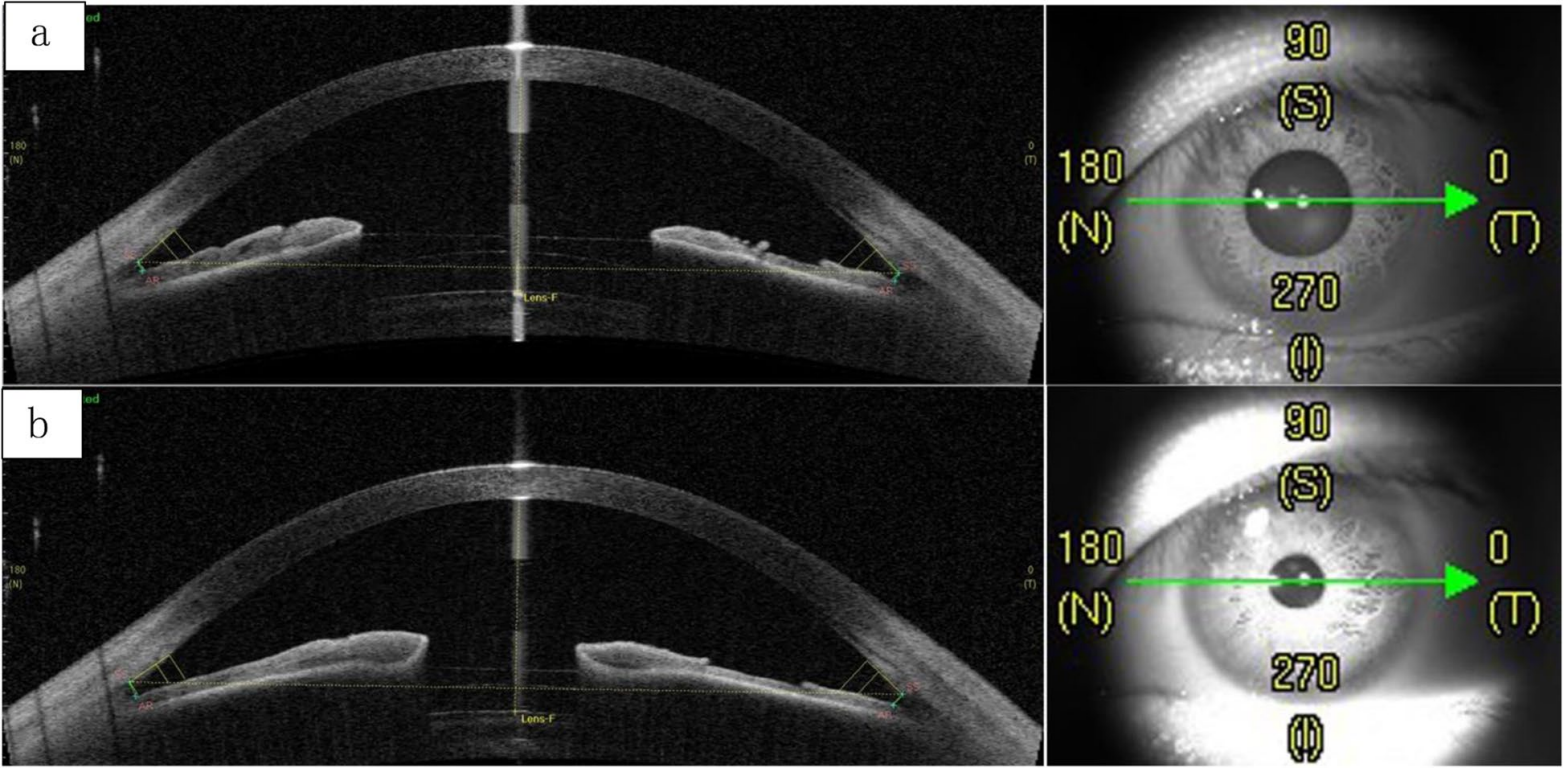
Changes in pupil and vault at different environmental light brightness. **a** mesopic condition; **b** photopic condition.

One eye from each patient was examined with Visante OCT (CASIA, Tomey). The anterior chamber depth, pupil size and postoperative vault were evaluated with Visante OCT under different lighting conditions 1 week after the operation. Scans were obtained in the horizontal meridian and each scan was measured three times. First, mesopic measurements were performed in a room luminance of 0.11 lx, which was monitored using a spectral irradiance sensor (SIS-20; China). Second, after the light reflex was induced by shining a penlight into the contralateral eye, photopic measurements were performed. When the ambient light intensity changed from 0.1 lx (Fig. 1a) to 5962.8 lx (Fig. 1b), the changes of pupil size and vault are shown in Fig. 1. In the measurement process, by changing the ambient light intensity, we could clearly see that the continuous movement of the pupil allowed the EVO ICL to get closer to the lens.

### Statistical analysis

The results are expressed as the mean ± standard deviation. The normality of the data was checked by Kolmogorov-Smirnov test. We applied the paired t test to compare postoperative vault, ACD and pupil size under different lighting conditions in both myopic and toric groups. For the comparison of vault changes and other measurements between myopic and toric groups, we performed independent t tests for continuous variables and x^2^ tests for categorical variables. The regression equation of the rate of vault change was obtained by using linear regression analysis. All data were analyzed with SPSS software (SPSS 19.0). Differences were considered statistically significant at *P* < 0.05.

## Results

**Fig. 2:**
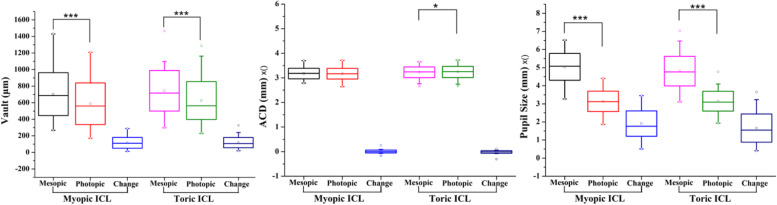
Comparisons of postoperative vault, anterior chamber depth and pupil size between photopic and mesopic conditions of eyes that were implanted with myopic or toric ICLs. Error bars represent the range of the standard deviation of the mean (***P < 0.001)

A summary of the toric and the myopic EVO ICL data is shown in Table 1. Postoperative vault, ACD, and pupil sizes of the eyes implanted with myopic and toric EVO ICLs under mesopic and photopic conditions are demonstrated in Table 2 and Fig. 2. The postoperative uncorrected distance visual acuity (UDVA) was 1.0 ± 0.2 and the spherical equivalent (SE) was + 0.25D ± 0.5D, respectively.

**Table 1:** Patient Data

Parameter	Myopic ICL	Toric ICL	***p***-value
No. of patients	68	60	
Gender			
Female	58	42	
Male	10	18	
Age (yrs)			
Mean	28.4 ± 6.0	26.4 ± 6.8	0.07
Range	18 to 48	18 to 41	
ACD (mm)			
Mean ± SD	3.18 ± 0.21	3.22 ± 0.23	0.15
Range	2.80 to 3.71	2.66 to 3.65	
Mean Power			
Sphercial	−10.1 ± 3.9 (− 27.0 to − 3.0)	−11.1 ± 4.4 (− 25.0 to − 1.0)	0.2
Cylindrical	−0.3 ± 0.5 (− 1.5 to 0.0)	− 2.2 ± 0.9 (− 4.5 to − 1.0)	<0.0001

*ICL* Implantable Collamer lens

**Table 2:** Postoperative Vault, Anterior Chamber Depth and Pupil Size Changes with Myopic and Toric ICL under Different Lighting Conditions

Characteristics	Myopic ICL		Toric ICL	
	Mesopic	Photopic	Mesopic	Photopic
ICLVault (μm)				
Mean ± SD	703.5 ± 259.9	587.8 ± 251.8	744.8 ± 244.7	626.2 ± 230.0
Range	266 to 1430	171 to 1209	297 to 1465	228 to 1287
△ (Mean/SD/range)	115.7 ± 64.9 (14 to 288) ±		118.1 ± 61.7 (19 to 326) to	
ACD (mm)				
Mean ± SD	3.17 ± 0.22	3.17 ± 0.22	3.23 ± 0.22	3.24 ± 0.23
Range	2.79 to 3.70	2.64 to 3.71	2.66 to 3.65	2.67 to 3.72
△ (Mean/SD/range)	0.01 ± 0.06 (− 0.17 to 0.26) ±		− 0.01 ± 0.05 (− 0.31 to 0.11) to	
Pupil Size (mm)				
Mean ± SD	5.04 ± 0.74	3.14 ± 0.57	4.80 ± 0.82	3.15 ± 0.55
Range	3.27 to 6.562	1.86 to 4.41	3.11 to 7.04	1.93 to 4.77
△ (Mean/SD/range)	1.91 ± 0.70 (0.50 to 3.45) ±		1.66 ± 0.78 (0.41 to 3.65) to	

*ICL* Implantable collamer lens, *ACD* Anterior chamber depth, △: Change; *SD* Standard deviation

27.50 ± 6.12

31.89 ± 6.17

− 4.38 ± 4.65

The eyes that were implanted with myopic and toric EVO ICLs showed significant differences in vault between mesopic and photopic conditions. In the eyes that were implanted with myopic EVO ICLs, the mean results for vault under mesopic and photopic conditions were 703.5 ± 259.9 μm and 587.8 ± 251.8 μm, respectively. Compared to mesopic conditions, a significant mean reduction of 115.7 ± 64.9 μm (95% confidence interval, *P* < 0.001) was found under photopic conditions.

In the eyes implanted with toric EVO ICLs, the mean results for vault under mesopic and photopic conditions were 744.8 ± 244.7 μm and 626.2 ± 230.0 μm, respectively, demonstrating a significant reduction in mean vault under photopic conditions (118.1 ± 61.7 μm; 95% confidence interval, *P* < 0.001). In all the eyes implanted with myopic EVO ICLs and toric EVO ICLs, vault change under mesopic and photopic was 116 ± 66 μm.

Change in ACD between mesopic and photopic conditions was not statistically significant with myopic EVO ICLs and slightly increased with toric EVO ICLs. In addition, there are no changes in ACD according to differing lighting conditions between both groups. As expected, the mean pupil sizes of eyes implanted with myopic and toric EVO ICLs were significantly larger under mesopic conditions. However, there were no significant difference in vault changes, ACD changes and pupil-size changes between the two groups under differing lighting conditions.

**Fig. 3:**
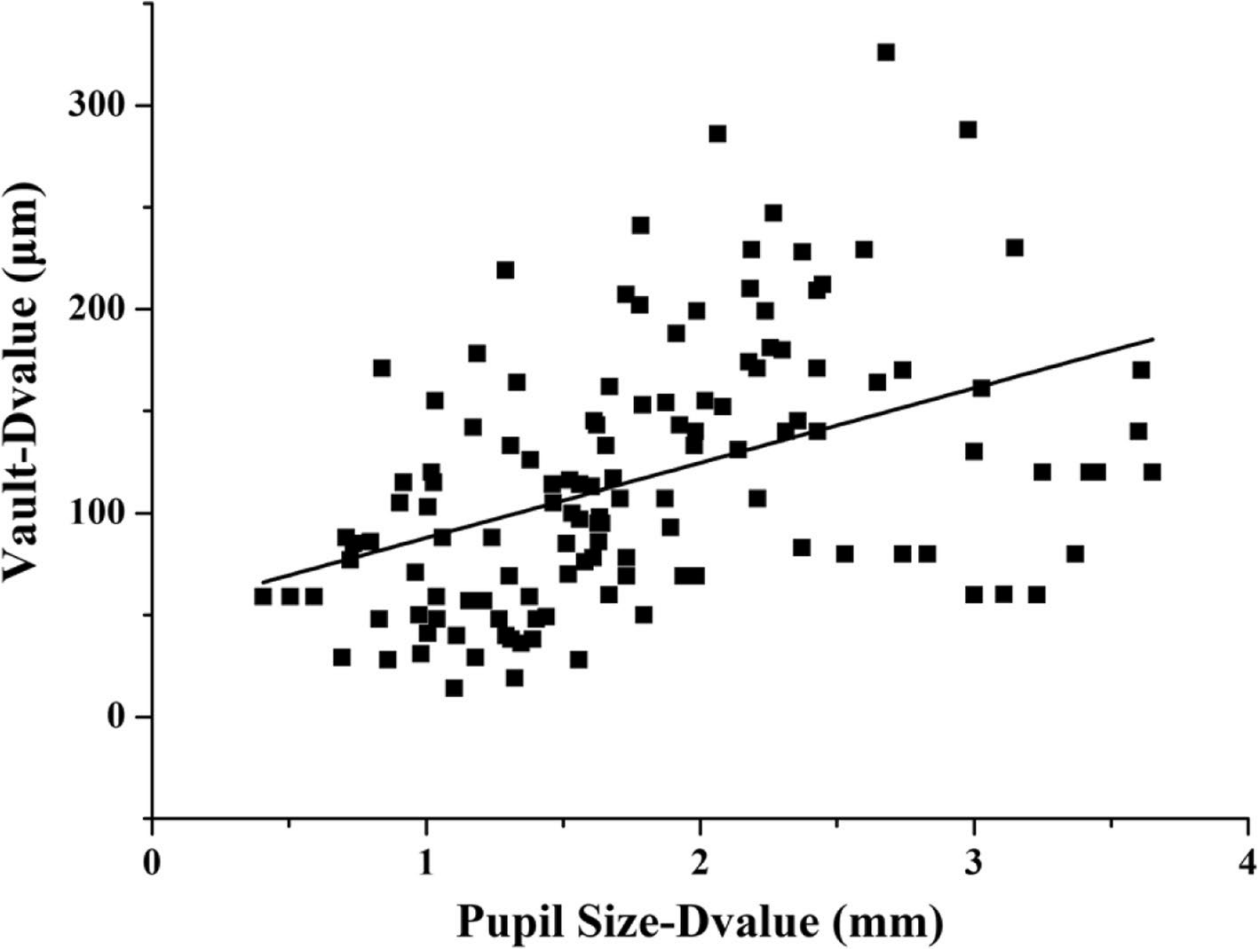
A scatterplot showing the vault change (Vault-Dvalue) relative to change of pupil size (Pupil size-D-value) under different lighting conditions

Figure 3 shows the relationship between the vault change (Vault-Dvalue) relative to change of pupil size (Pupil size-D-value) under different lighting conditions. The regression equation of the vault change was obtained by using linear regression analysis: Vault-Dvalue = 36.67* Pupil Size-Dvalue (μm) + 51.14, and the adjusted r^2^ was 0.182. The vault change was significantly increased with an increase of the change of pupil size (*p* < 0.01).

**Fig. 4:**
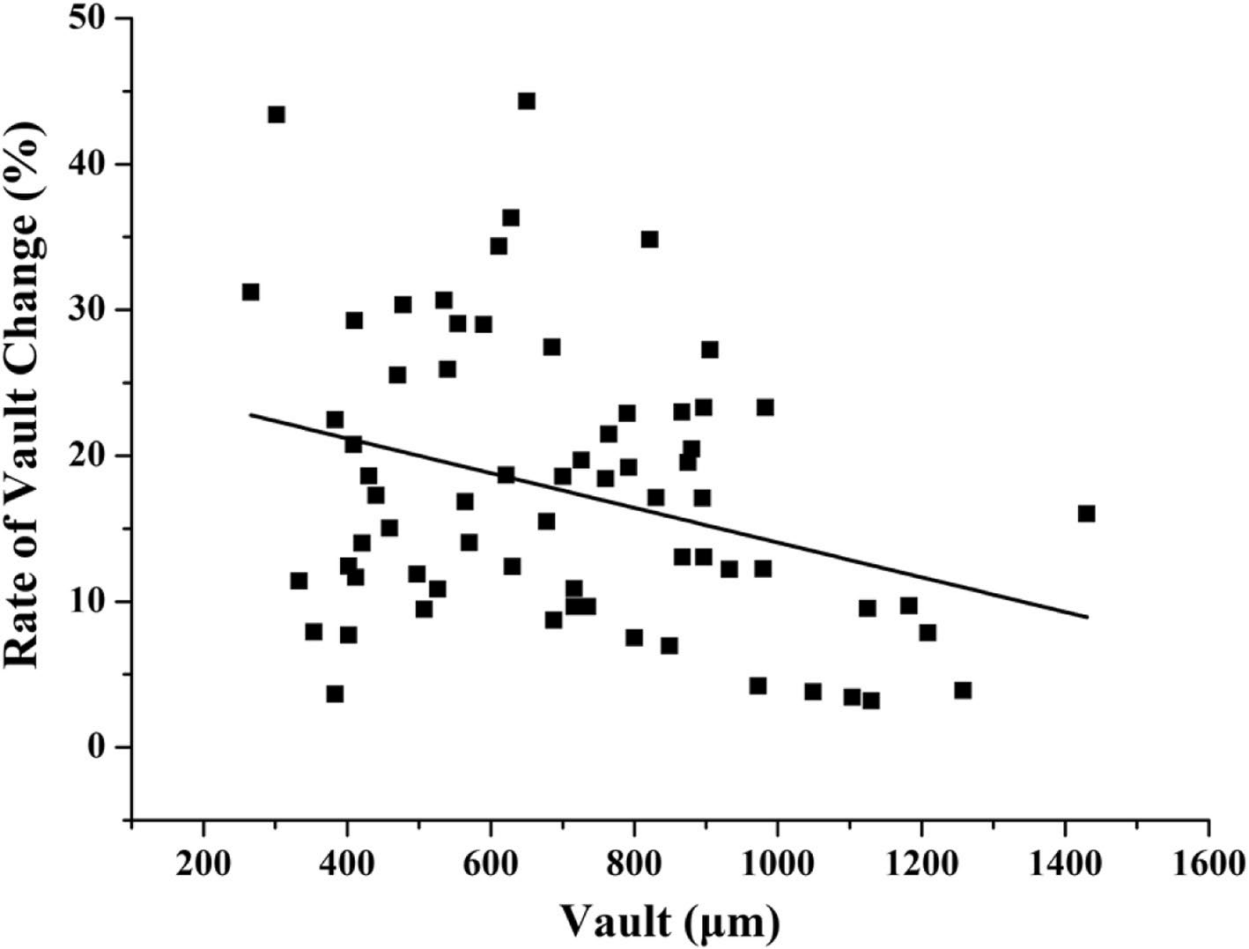
A scatterplot showing the rate of vault change relative to mesopic vault

Figure 4 shows the relationship between the rate of vault change and mesopic vault (baseline value). The regression equation of the rate of vault change was obtained by using linear regression analysis: Rate of vault change = − 1.04 E^− 5^*Vault (μm) + 0.246, and the adjusted r^2^ was 0.081. The rate of vault change was significantly decreased with an increase of mesopic vault (*p* < 0.01).

## Discussions

At present, the commonly used method for measuring vault (distance between the posterior ICL surface and anterior crystalline lens surface) in clinical practice is static value measurement, such as subjective judgment in slit-lamp examinations, high frequency ultrasound biomicroscopy, scheimpflug imaging instrument, and anterior segment OCT. The ideal vault after ICL implantation is generally 0.5 to 1.5 times the corneal thickness (CT, 250 to 750 μm). However, the vault after the ICL implantation is dynamic due to the human accommodation.

Lindland [11] et al. found that the vault decreased 40 ± 60 μm from mesopic condition (2 lx) to photopic condition (257 lx). Petternel [12] et al. found that the vault decreased 73 ± 50 μm from mesopic condition to photopic condition. Lee [6] et al. found that the vault decreased 147.5 ± 59.5um under pen light. Gonzale-Lopez [13] et al. found that the vault decreased 167 ± 70 μm from photopic condition (18,500 lx) to mesopic condition (0.5 lx). Sayaka [14] et al. found that the vault change decreased 55 ± 35 μm from mesopic condition (4 lx) to photopic condition (400 lx). The vault in our study decreased 116 ± 66 μm from mesopic condition (0.1 l lux) to photopic condition (5962.8 lx).

The difference of valut change in different studies may be absribed to the follow reasons: First, ICL used in Lindland [11] et al. and Petternel [12] et al. studies were ICL without central flow while the ICL used in Lee [6] et al., Gonzale-Lopez [13] et al., Sayaka [14], et al., and our studies were ICL with central flow. Preclinical studies demonstrated that the 0.36 mm central port incorporated in the design of ICL provides sufficient aqueous flow to maintain normal fluid dynamics in the eye and it improves the circulation of aqueous around the crystalline lens [15–20]. Second, all these studies were conducted in different lighting conditions. Our study used strong stimulation with 5962.8 lx light intensity under photopic condition. When induced by stronger light, the pupil shrank more significantly. The iris tension pushed the ICL downward towards the lens more significantly, resulting in higher vault change.

In accordance to what Lindland [11] et al. discovered, we also did not find any significant differences in vault changes, ACD changes and pupil-size changes between myopic and toric groups under different lighting conditions either. Moreover, it is worth noting that the rate of vault change under different lighting conditions was significantly decreased with increased mesopic vault (baseline value). When induced by a strong light, the pupil shrank, the iris tension pushed the ICL downward towards the lens and it caused the ICL to bend in order to adapt to the posterior surface of the iris. Such movement enabled the aqueous humor to flow through the lateral and central holes. Within the normal mesopic vault, the fluctuation of humor flow increases the ICL’s forwards and backwards movement, along with the effect that movement of ICL and anterior chamber also enabled the aqueous humor to flow through the lateral and central holes. However, too high a mesopic vault would constrict the posterior movement of pupil, thus influence, restrict the posterior movement of the ICL. More than that, too low a mesopic vault has a big rate of vault change, which may cause the contact of ICL with crystalline lens in photopic state, resulting to the risk of opacification of crystalline lens.

The findings of the study suggest that, for patient with high vault (in addition to anterior chamber angle), we should be more careful and must perform checks to see if there is any iris dysfunction that is present; if necessary, an explant or an exchange to a smaller sized ICL should be done. For patients with low vault, clinical follow-up and monitoring is necessary, especially in photopic state.

The advantage of this study is that for all the included eyes, the vault was measured the same time after operation, which eliminated the time span on the change of vault. It has been reported that the vault is most unstable in the first 3 days after operation, but it can reach a relatively stable range 1 week postoperative [21]. The limitation of this study is that the postoperative long-term follow-up of vault is not considered in the analysis. Future studies will evaluate whether the change of the vault will be affected within a long passage of time.

## Conclusions

Too low a mesopic vault has a big rate of vault change, which may cause the contact of ICL with crystalline lens in photopic state, resulting to the risk of opacification of crystalline lens. Too high a mesopic vault would constrict the posterior movement of pupil, thus influence, restrict the posterior movement of the ICL. The findings of the study suggest that, for patient with high vault (in addition to anterior chamber angle), we should be more careful and must perform checks to see if there is any iris dysfunction that is present; if necessary, an explant or an exchange to a smaller sized ICL should be done. For patients with low vault, clinical follow-up and monitoring in photopic state is needed; if necessary, an explant or an exchange to a bigger sized ICL should be done.

## Data Availability

The datasets generated during and analyzed during the current study are not publicly available due to privacy and ethical restrictions but are available from the corresponding author on reasonable request.
